# Red cell distribution width, anemia, and lower-extremity physical function among rural-dwelling older adults

**DOI:** 10.1007/s40520-022-02187-9

**Published:** 2022-08-09

**Authors:** Ziying Jiang, Xiaolei Han, Yongxiang Wang, Tingting Hou, Yi Dong, Xiaodong Han, Anna-Karin Welmer, Lenore J. Launer, Yifeng Du, Chengxuan Qiu

**Affiliations:** 1 Department of Neurology, Shandong Provincial Hospital, Cheeloo College of Medicine, Shandong University, No. 324 Jingwuweiqi Road, Jinan, Shandong China; 2 Department of Neurology, Shandong Provincial Hospital, Affiliated to Shandong First Medical University, No. 324 Jingwuweiqi Road, Jinan, Shandong China; 3 Aging Research Center and Division of Physiotherapy, Department of Neurobiology, Care Sciences and Society, Karolinska Institutet, Stockholm, Sweden; 4 Women´S Health and Allied Health Professionals Theme, Medical Unit Medical Psychology, Karolinska University Hospital, Stockholm, Sweden; 5 Laboratory of Epidemiology and Population Sciences, National Institute On Aging, National Institutes of Health, Bethesda, MD USA; 6 Aging Research Center and Center for Alzheimer Research, Department of Neurobiology, Care Sciences and Society, Karolinska Institutet-Stockholm University, Stockholm, Sweden

**Keywords:** Red cell distribution width, Anemia, Physical function, Elderly, Population-based study

## Abstract

**Background:**

Elevated red cell distribution width (RDW) has been associated with degenerative conditions in aging.

**Aims:**

We aimed to evaluate the associations of RDW and anemia with lower-extremity physical function among rural-dwelling older adults.

**Methods:**

This population-based cross-sectional study included 5093 rural residents (age ≥ 60 years, 57.3% women) who participated in the MIND-China Study in Shandong. Data were collected via face-to-face interviews, clinical examinations, and laboratory tests. RDW was categorized according to quartiles and the lower-extremity physical function was assessed using the Short Physical Performance Battery (SPPB),

**Results:**

Multiple linear regression analyses suggested that the fourth quartile of RDW (vs. first quartile) was associated with lower SPPB summary score (β-coefficient – 0.38; 95% CI – 0.58 to – 0.18) and lower scores in balance test (– 0.09; – 0.17 to – 0.01), chair stand test (– 0.17; – 0.27 to – 0.07), and walking speed test (– 0.12; – 0.19 to − 0.05). Anemia was associated with a multiple-adjusted β-coefficient of – 0.34 (– 0.52 to – 0.16) for SPPB summary score. Stratified analysis by anemia showed that there was a linear association between RDW and SPPB in individuals without anemia but a J-shaped association in individuals with anemia.

**Discussion:**

This large-scale population-based study revealed the associations of high RDW and anemia with poor lower-extremity physical function among rural-dwelling Chinese older adults. These findings suggest that an elevated RDW might be a biochemical marker for poor lower-extremity physical function among older adults.

**Conclusions:**

Anemia and an elevated RDW are associated with poor performance in lower-extremity physical function among rural-dwelling Chinese older adults.

## Introduction

Lower-extremity physical function deteriorates as people age, which significantly affects quality of life of older people [1]. The lower-extremity physical performance reflects the changes of the individual’s anatomical and physiological system. Impairment in lower-extremity physical function could indicate poor general health status and predict subsequent adverse health outcomes, such as falls [2], hospitalization [3], disability [4], and mortality [5]. Given that therapeutic approaches to postponing physical function decline in elderly people remain limited, exploring modifiable risk factors will facilitate the development of preventive and therapeutic interventions to delay physical impairment and disability.

Red cell distribution width (RDW), a part of the standard complete blood count (CBC), is a parameter of the heterogeneity of the circulating erythrocyte volume and is conventionally used to indicate anisocytosis in blood circulation [6]. It is customarily calculated as a percentage of the standard deviation of red blood cell volume divided by the mean corpuscular volume (MCV). Normal value of RDW ranges between 12 and 15%, although it slightly fluctuates depending on the study populations and hematology analyzer [7]. Traditionally, RDW is applied to classifying anemias in conjunction with MCV. Elevated RDW (heterogeneous red blood cell size) is generally associated with vitamin B_12_, folate deficiency anemia, iron deficiency anemia, and sideroblastic anemia, whereas normal RDW (homogeneous red blood cell size) is generally associated with aplastic anemia, thalassemia heterozygosity, and anemia due to chronic diseases. In recent years, numerous studies have shown that an elevated RDW is associated with mortality [8], cardiovascular disease [9], frailty [10], and other aging-related degenerative outcomes [11] among elderly people.

In addition, previous population-based studies have shown that anemia is associated with impaired physical function and accelerated declines in physical performance among older adults [12, 13]. The cross-sectional study of community-dwelling older adults in Taiwan suggested that a high RDW (≥ 15.7%) was associated with an increased risk of frailty [10]. However, population-based studies have rarely investigated the association between RDW and geriatric lower-extremity physical function among older adults. Previous studies also have indicated that the associations between RDW and health outcomes vary with anemia status [14, 15]. This suggests that anemia conditions should be taken into consideration when examining the relationship between RDW and physical function in older adults. Thus, the main objectives of this population-based cross-sectional study were (1) to examine the relationships of RDW and anemia with lower-extremity physical function among Chinese older adults living in rural communities, and further (2) to further explore the relationships between RDW and lower-extremity physical function by anemia status.

## Methods

### Study design and participants

This population-based cross-sectional study used data collected from participants in the baseline examination of the Multimodal Interventions to Delay Dementia and Disability in rural China (MIND-China) in Yanlou Town, Yanggu County in Shandong Province, conducted by Shandong Provincial Hospital in collaboration with the Yanlou Town Health Center. As a participating project in the World-Wide FINGERS Network (a global network for dementia risk reduction and prevention) [16], MIND-CHINA was aimed to test the effects of multidomain interventions (e.g., lifestyle and nutritional guidelines, physical exercise, cognitive training, and improvement in the management of major cardiovascular risk factors) on cognitive decline and onset of dementia and disability among rural-dwelling elderly people [17]. MIND-China targeted older adults who were aged 60 years or older and living in the 52 communities (villages) in Yanlou Town. Baseline assessments for MIND-China were performed between March and September 2018 in combination with the annual health check-up for local elderly residents, as previously reported [18]. In total, 5765 participants undertook the baseline survey. Of these, we excluded 667 persons who did not take part in physical function tests due to logistic reasons. We further excluded five participants who had missing information on RDW, leaving 5093 (88.3%) participants for the current analysis.

### Data collection and definition

Trained staff collected data via face-to-face interviews, physical examinations, neuropsychological testing, and laboratory tests, following a structured questionnaire, as previously reported [18, 19]. Briefly, the data included socio-demographics, lifestyles (e.g., alcohol use, smoking, and physical activity), medical conditions (e.g., hypertension, diabetes, coronary heart disease, and stroke), use of medications, and cognitive and functional assessments. Medications were classified and coded following the Anatomical Therapeutic Chemical (ATC) classification system [20]. Based on self-reported information, smoking status was categorized into current, former, or never smoking; alcohol consumption as yes versus no; and physical activity as moderate-to-high activity, low activity, or inactivity [19].

Body mass index (BMI) was defined as weight divided by height squared (kg/m^2^). After a 5-min rest, arterial blood pressure was measured on the right upper arm using an electronic sphygmomanometer. Total cholesterol, triglycerides, and fasting plasma glucose were measured using the enzymatic methods by an automatic biochemical analyzer. Hypertension was defined as systolic pressure ≥ 140 mmHg or diastolic pressure ≥ 90 mmHg or current use of antihypertensive medications (ATC codes C02, C03, and C07–09); diabetes as fasting blood glucose ≥ 7.0 mmol/L, having a self-reported history of diabetes or current use of hypoglycemic medications (ATC code A10); and dyslipidemia as serum total cholesterol ≥ 6.22 mmol/l or low-density lipoprotein cholesterol ≥ 4.14 mmol/l or triglyceride ≥ 2.26 mmol/l or high-density lipoprotein cholesterol < 1.04 mmol/l or (5) current use of lipid-regulating medications (ATC code C10) [21]. Stroke and coronary heart disease were ascertained via self-reported history and clinical examination. Impaired renal function was defined as the creatinine-based estimated glomerular filtration rate (eGFR) < 60 ml/min/1.73 m^2^ [22]. The 15-item Geriatric Depressive Scale (GDS-15) was used to assess depressive symptoms. The presence of depressive symptoms was defined as having a GDS-15 score ≥ 5[23].

### Measurements of RDW and hemoglobin and definition of anemia

Peripheral blood samples were collected in a tube with 2 mL ethylenediaminetetraacetic acid (EDTA) after an overnight fast. A Mindray BC-6800 automated hematology analyzer (Mindray Medical International Ltd., Shenzhen, CHN) was used to assay the complete blood count, such as RDW and hemoglobin concentrations, in the laboratory of Yanlou Town Health Center. Anemia was diagnosed according to the WHO criteria [24]: hemoglobin < 13 g/dl for men and < 12 g/dl for women.

### Assessment of lower-extremity physical performance

The Short Physical Performance Battery (SPPB), consisting of a series of physical functional tests (balance, chair stand, and walking speed), was used to evaluate lower-extremity physical function. For the balance test, participants were required to remain standing in three increasingly difficult positions (side-by-side, semi-tandem, and full-tandem positions) each for ten seconds. For the chair stand test, participants were required to cross arms over their chest and complete five consecutive times sit-to-stand from a chair as quickly as possible. For the walking speed test, participants were required to walk four meters at a usual pace and repeat twice. Each of these three physical performance tests was converted into a score ranging from 0 to 4. Then, these three scores were added up to yield the SPPB summary score (range 0– 12), with higher values representing better physical performance. Previous studies have demonstrated that SPPB can predict falls, disability, hospitalization, and mortality among older adults and the instrument has been frequently used for measuring physical performance among elderly people [25– 28].

### Statistical analysis

RDW was analyzed both as a continuous variable and as a categorical variable of quartiles (Q1: < 13.1%, Q2: 13.1% to 13.5%, Q3: 13.6% to 14.0%, and Q4: > 14.0%). Descriptive statistics of participants were presented by RDW quartiles, in which we reported mean (standard deviation, SD) for continuous variables and frequencies (%) for categorical variables. We used one-way analysis of variance (ANOVA) and post hoc ANOVA analysis for comparison of continuous variables and chi-squared test for categorical variables. Multiple linear regression analyses were performed to estimate β-coefficient and 95% confidence interval (CI) of SPPB score associated with RDW levels and anemia. We used the restricted cubic Spline regression analysis to test the potential nonlinear relationship. We controlled for multiple potential confounders in two different models when examining their associations: model 1 was adjusted for age, sex, and education; and model 2 was additionally adjusted for alcohol use, smoking, BMI, physical activity, hypertension, diabetes, dyslipidemia, impaired renal function, a history of stroke and coronary heart disease, and the presence of depressive symptoms. We further tested statistical interaction between RDW quartile groups and anemia on physical performance by simultaneously including the two independent variables and their cross-product term in the same model. When a statistical interaction was detected (*P* for interaction < 0.05), stratified analysis by anemia was further performed. In stratified analysis, RDW was categorized into quartile groups according to anemia strata. Multicollinearity was checked to be satisfied in all models using the variance inflation factor values. All statistical analyses were performed using IBM SPSS Statistics for Windows, Version 22.0 (IBM Corp., Armonk, NY, USA), except the restricted cubic spline regression analysis where the R package for Windows (version 4.0.2, R Foundation for Statistical Computing, Vienna, Austria) was used. We considered a two-tailed *P* < 0.05 to be statistically significant.

## Results

Table 1 presents characteristics of the study participants across quartiles of RDW. The mean age of all the 5093 participants was 70.5 (SD = 5.5; range, 60– 93) years, and 57.3% were women. The mean RDW level was 13.6% (SD = 0.9; range, 11.0– 29.5%) and 995 (19.5%) were defined to have anemia. The mean SPPB summary score was 9.3 (SD = 2.8; range, 0– 12). Compared to participants with lower RDW, those with higher RDW were significantly older, had lower BMI and were more likely to be female and to have prevalent hypertension, diabetes, impaired kidney function, and anemia (*P* < 0.05). With an increase in RDW, there was a trend towards a decrease in the mean SPPB summary score as well as the scores of balance, chair stand, and walking speed tests (*P* < 0.001). The post hoc ANOVA analysis showed that the SPPB summary score was significantly lower in people with Q4 of RDW than those with Q1, Q2, and Q3 of RDW, respectively (*P* < 0.01), whereas there were no significant differences in the mean SPPB summary score among groups of persons with Q1, Q2, and Q3 of RDW (*P* > 0.05). There were similar patterns of differences in the mean scores of balance, chair stand, and walking speed tests among the quartile groups of RDW.

**Table 1:** Characteristics of the study participants by quartiles of red cell distribution width

		Red cell distribution width, quartiles				
Characteristics	Total sample (n = 5093)	Q1 < 13.1% (n = 1289)	Q2 13.1%-13.5% (n = 1427)	Q3 13.6%-14.0% (n = 1277)	Q4 > 14.0% (n = 1100)	*P*^a^
Socio-demographic factors						
Age (years), mean (SD)	70.5 (5.5)	69.7 (5.1)	70.1 (5.5)	70.4 (5.5)	71.9 (5.8)	< 0.001
Female, n (%)	2917 (57.3)	783 (60.7)	807 (56.6)	737 (57.7)	590 (53.6)	0.005
Education (years), mean (SD)	3.2 (3.4)	3.3 (3.6)	3.3 (3.6)	3.3 (3.5)	3.2 (3.4)	0.833
Lifestyle factors						
Alcohol consumption^b^, n (%)	2004 (39.4)	486 (37.8)	564 (39.7)	495 (38.8)	459 (41.8)	0.222
Smoking^b^, n (%)						0.144
Never	3466 (68.1)	914 (70.9)	962 (67.5)	870 (68.1)	720 (65.5)	
Former	543 (10.7)	122 (9.5)	153 (10.7)	133 (10.4)	135 (12.3)	
Current	1083 (21.3)	253 (19.6)	311 (21.8)	274 (21.5)	245 (22.3)	
Physical activity^b^, *n* (%)						0.264
Inactive	1819 (35.8)	470 (36.5)	539 (37.8)	429 (33.6)	381 (34.7)	
Low	608 (11.9)	146 (11.3)	161 (11.3)	156 (12.2)	145 (13.2)	
Moderate-to-high	2661 (52.3)	673 (52.2)	726 (50.9)	691 (54.2)	571 (52.1)	
Clinical factors						
Body mass index^b^ (kg/m^2^), mean (SD)	24.9 (3.8)	24.8 (3.6)	25.0 (3.6)	25.1 (3.8)	24.7 (4.1)	0.015
Hypertension^b^, n (%)	3345 (66.3)	895 (70.2)	948 (67.1)	830 (65.5)	672 (61.5)	< 0.001
Diabetes mellitus, n (%)	735 (14.4)	221 (17.1)	211 (14.8)	171 (13.4)	132 (12.0)	0.003
Dyslipidemia, n (%)	1216 (23.9)	316 (24.5)	361 (25.3)	293 (22.9)	246 (22.4)	0.276
Stroke, n (%)	816 (16.0)	182 (14.1)	247 (17.3)	208 (16.3)	179 (16.3)	0.148
Coronary heart disease, n (%)	1066 (20.9)	271 (21.0)	274 (19.2)	286 (22.4)	235 (21.4)	0.224
Impaired kidney function, n (%)	501 (9.8)	93 (7.2)	143 (10.0)	125 (9.8)	140 (12.7)	< 0.001
GDS-15 score ≥ 5^b^, n (%)	488 (9.8)	140 (11.0)	137 (9.8)	122 (9.7)	89 (8.4)	0.226
Anemia, n (%)	995 (19.5)	167(13.0)	204 (14.3)	257 (20.1)	367 (33.4)	< 0.001
Physical performance						
SPPB summary score, mean (SD)	9.3 (2.8)	9.5 (2.6)	9.4 (2.6)	9.3 (2.8)	8.8 (3.0)	< 0.001
Balance test score, mean (SD)	3.4 (1.1)	3.5 (1.0)	3.4 (1.0)	3.4 (1.1)	3.3 (1.2)	< 0.001
Chair stand test score, mean (SD)	2.6 (1.3)	2.7 (1.3)	2.7 (1.3)	2.6 (1.3)	2.4 (1.4)	< 0.001
Walking speed test score, mean (SD)	3.3 (1.0)	3.4 (0.9)	3.3 (0.9)	3.3 (1.0)	3.1 (1.1)	< 0.001

*GDS-15* 15-item Geriatric Depressive Scale, *SD* standard deviation, *SPPB* Short Physical Performance Battery

^a^*P* value was for the test of differences among the quartile groups of red cell distribution width

^b^The numbers of people with missing values were 12 in alcohol consumption, 1 in smoking, 5 in physical activity, 31 in body mass index, 46 in hypertension, and 108 in GDS-15 score

Table 2 shows the association between RDW, anemia, and lower-extremity physical performance from the multivariable linear regression models. In the fully adjusted models, people with anemia had a lower SPPB summary score compared to those without anemia (multiple-adjusted β-coefficients = – 0.34; 95% CI – 0.52 to – 0.16). When RDW was analyzed as a continuous variable, per 1-unit (%) increment in RDW was significantly associated with a reduction of approximately 0.19 points in SPPB summary score, even controlling for multiple potential confounders in model 2. When RDW was analyzed as quartiles, the upper RDW quartile was significantly associated with a reduced β-coefficients of SPPB summary score, in a dose-response manner (*P* for linear trend < 0.001). Compared to participants with the lowest quartile (Q1), those with the highest quartile of RDW level (Q4) had a significantly lower SPPB summary score (multiple-adjusted *β* = – 0.38; 95% CI – 0.58 to – 0.18), but there was a non-significantly reduced SPPB score in those with Q3 (multiple-adjusted β = – 0.15; 95% CI = – 0.34 to – 0.04) and those with Q2 of RDW level (multiple-adjusted β = – 0.03; 95% CI = – 0.21 to – 0.16). Similarly, anemia and a higher RDW were significantly associated with lower scores in balance test, chair stand test, and walking speed test (*P* < 0.05).

**Table 2:** Associations of anemia and red cell distribution width with Short Physical Performance Battery summary and subtest scores

Anemia or RDW	No. of	SPPB summary score		SPPB balance score		SPPB chair stand score		SPPB walking speed score	
	subjects								
		β-coefficient (95% CI)		β-coefficient (95% CI)		β-coefficient (95% CI)		β-coefficient (95% CI)	
		Model 1^a^	Model 2^a^	Model 1^a^	Model 2^a^	Model 1^a^	Model 2^a^	Model 1^a^	Model 2^a^
Anemia									
No	4098	0.00(Ref.)	0.00(Ref.)	0.00(Ref.)	0.00(Ref.)	0.00(Ref.)	0.00(Ref.)	0.00(Ref.)	0.00(Ref.)
Yes	995	– 0.35 (– 0.54,– 0.17)	– 0.34 (– 0.52,– 0.16)	– 0.10 (– 0.18, – 0.03)	– 0.09 (– 0.16, – 0.02)	– 0.16 (– 0.25,– 0.07)	– 0.16 (– 0.25,– 0.07)	– 0.09 (– 0.15,– 0.02)	– 0.09 (– 0.16,– 0.03)
RDW (%), continuous	5093	– 0.20 (– 0.28,– 0.12)	– 0.19 (– 0.27,– 0.12)	– 0.05 (– 0.09, – 0.02)	– 0.05 (– 0.08, – 0.02)	– 0.08 (– 0.12,– 0.04)	– 0.08 (– 0.12,– 0.04)	– 0.07 (– 0.10, – 0.04)	– 0.06 (– 0.09,– 0.04)
RDW (%), Quartiles^b^									
Q1	1289	0.00(Ref.)	0.00(Ref.)	0.00(Ref.)	0.00(Ref.)	0.00(Ref.)	0.00(Ref.)	0.00(Ref.)	0.00(Ref.)
Q2	1427	– 0.05 (– 0.25, 0.14)	– 0.03 (– 0.21, 0.16)	– 0.02 (– 0.10, 0.05)	– 0.01 (– 0.08, 0.07)	– 0.03 (– 0.12, 0.07)	– 0.03 (– 0.12, 0.07)	– 0.01 (– 0.07, 0.06)	0.01 (– 0.06, 0.07)
Q3	1277	– 0.13 (– 0.33, 0.07)	– 0.15 (– 0.34, 0.04)	– 0.01 (– 0.09, 0.07)	– 0.02 (– 0.10, 0.06)	– 0.09 (– 0.19, 0.01)	– 0.10 (– 0.20,– 0.01)	– 0.03 (– 0.10, 0.04)	– 0.03 (– 0.09, 0.04)
Q4	1100	– 0.38 (– 0.59, – 0.17)	– 0.38 (– 0.58,– 0.18)	– 0.08 (– 0.17, – 0.01)	– 0.09 (– 0.17, – 0.01)	– 0.17 (– 0.27, – 0.07)	– 0.17 (– 0.27,– 0.07)	– 0.13 (– 0.20, – 0.05)	– 0.12 (– 0.19,– 0.05)
*P* for trend		<0.001	<0.001	0.087	0.049	0.001	<0.001	0.001	0.001

*CI* confidence interval, *RDW* red cell distribution width, *SPPB* Short Physical Performance Battery

^a^Model 1 was adjusted for age, sex, education; Model 2 was additionally adjusted for alcohol use, smoking, physical activity, body mass index, hypertension, diabetes mellitus, dyslipidemia, coronary heart disease, stroke, impaired renal function, depressive symptoms

^b^The cut-offs of RDW quartiles were < 13.1% (Q1), 13.1– 13.5% (Q2), 13.6– 14.0% (Q3), and > 14.0% (Q4)

We detected a statistical interaction of anemia with RDW levels on the SPPB summary score in model 2 (*P* for anemia × first RDW quartile interaction = 0.01). We further examined the association between RDW and SPPB score by anemia strata. In the stratified analysis, among participants without anemia per 1-unit (%) increment in RDW was significantly associated with a reduction of approximately 0.21 points in SPPB summary score, even when controlling for multiple potential confounders in model 2. A similar dose– response relationship between higher RDW quartiles and the lower SPPB summary score was confirmed (*P* = 0.001) (Table 3). In addition, there were similar linear relationships between RDW levels and scores of chair stand test and walking speed test among participants without anemia (*P* < 0.01). By contrast, among participants with anemia there was no significant linear association between RDW and SPPB summary score and subtest scores (*P* > 0.05); instead, the restricted cubic spline regression analysis suggested a nonlinear relationship between RDW quartiles and SPPB summary score, with the highest SPPB summary score being in the second RDW quartile. Thus, we used the second quartile of RDW as a reference group in the analysis of participants with anemia. The analysis showed an inverted J-shaped association, with the first and the fourth quartiles of RDW being marginally or significantly associated with lower scores in SPPB tests (Table 3).

**Table 3:** Associations of red cell distribution width with Short Physical Performance Battery summary and subtest scores among older adults by anemia

RDW (%),	No. of	SPPB summary score		SPPB balance score		SPPB chair stand score		SPPB walking speed score	
quartiles	subjects	β-coefficient (95% CI)		β-coefficient (95% CI)		β-coefficient (95% CI)		β-coefficient (95% CI)	
		Model 1^a^	Model 2^a^	Model 1^a^	Model 2^a^	Model 1^a^	Model 2^a^	Model 1^a^	Model 2^a^
Anemia, no^b^									
Q1	1122	0.00(Ref.)	0.00(Ref.)	0.00(Ref.)	0.00(Ref.)	0.00(Ref.)	0.00(Ref.)	0.00(Ref.)	0.00(Ref.)
Q2	847	– 0.13 (– 0.35, 0.09)	-0.15 (-0.36, 0.07)	– 0.04 (– 0.13, 0.05)	– 0.04 (– 0.12, 0.05)	– 0.06 (– 0.17, 0.06)	– 0.07 (– 0.18, 0.04)	– 0.03 (– 0.11, 0.05)	– 0.04 (– 0.11, 0.04)
Q3	981	– 0.15 (– 0.37, 0.06)	– 0.14 (– 0.34, 0.07)	– 0.03 (– 0.11, 0.05)	– 0.02 (– 0.11, 0.06)	– 0.09 (– 0.20, 0.02)	– 0.09 (-0.20, 0.01)	– 0.03 (– 0.11, 0.04)	– 0.02 (– 0.09, 0.05)
Q4	1148	– 0.37 (– 0.57, – 0.16)	– 0.36 (– 0.56, – 0.17)	– 0.04 (– 0.12, 0.05)	– 0.03 (– 0.11, 0.05)	– 0.22 (– 0.32, – 0.12)	– 0.23 (– 0.33, – 0.12)	– 0.11 (– 0.18, – 0.04)	– 0.11 (– 0.18, – 0.04)
*P* for trend		0.001	0.001	0.442	0.507	<0.001	<0.001	0.004	0.007
Anemia, yes^c^									
Q1	277	– 0.38 (– 0.87, 0.10)	– 0.40 (– 0.87, 0.06)	– 0.10 (– 0.30, 0.10)	– 0.09 (– 0.29, 0.10)	– 0.19 (– 0.42, 0.04)	– 0.21 (– 0.44, 0.02)	– 0.09 (– 0.26, 0.07)	– 0.10 (– 0.26, 0.07)
Q2	240	0.00(Ref.)	0.00(Ref.)	0.00(Ref.)	0.00(Ref.)	0.00(Ref.)	0.00(Ref.)	0.00(Ref.)	0.00(Ref.)
Q3	251	– 0.43 (– 0.92, 0.07)	– 0.53 (– 1.01, – 0.05)	– 0.23 (– 0.43, – 0.02)	– 0.28 (– 0.48, – 0.08)	– 0.13 (– 0.37, 0.10)	– 0.16 (– 0.39, 0.08)	– 0.07 (– 0.24, 0.10)	– 0.09 (– 0.26, 0.08)
Q4	227	– 0.56 (– 1.07, – 0.04)	– 0.64 (– 1.14, – 0.14)	– 0.21 (– 0.42, 0.00)	– 0.26 (– 0.47, – 0.05)	0.11 (– 0.36, 0.13)	– 0.14 (– 0.38, 0.11)	– 0.23 (– 0.41, – 0.05)	– 0.24 (– 0.422, – 0.07)

*CI* confidence interval,*RDW* red cell distribution width, *SPPB* Short Physical Performance Battery

^a^Model 1 was adjusted for age, sex, education; Model 2 was additionally adjusted for alcohol use, smoking, physical activity, body mass index, hypertension, diabetes mellitus, dyslipidemia, coronary heart disease, stroke, impaired renal function, depressive symptoms

^b^The cut-offs of RDW quartiles were < 13.1% (Q1), 13.1– 13.4% (Q2), 13.5– 13.7% (Q3), and > 13.7% (Q4)

^c^The cut-offs of RDW quartiles were < 13.3% (Q1), 13.3– 13.7% (Q2), 13.8– 14.3% (Q3), and > 14.3% (Q4)

## Discussion

The main findings from this large-scale population-based cross-sectional study of rural residents aged 60 years and older can be summarized into two points. First, our study revealed that high RDW and anemia were associated with poorer performance in lower-extremity physical function, independent of multiple potential confounders. Second, the association between RDW levels and SPPB scores varies by anemia status, such that there was a linear relationship between RDW levels and the likelihood of impaired lower-extremity physical function among individuals without anemia, but an inverted J-shaped relationship in individuals with anemia.

So far, very few population-based studies have investigated the association between RDW and physical performance in older adults. The prospective Osteoporotic Fractures in Men study (age ≥ 65 years) found that a higher RDW was associated with weaker lower-extremity muscle strength and slower walking speed at baseline[11]. Our study complements previous results by showing that RDW could be a potential biomarker for impaired performance in lower-extremity physical function among an older population in rural China. The overall prevalence of anemia was 19.5% in our study population, which was broadly consistent with the previous reports from China[29]. Previously, the population-based cross-sectional studies have investigated the association between anemia and physical performance among older adults [12], which yielded results that were generally comparable with our findings. Similarly, the cross-sectional data from the Italian InChianti Study of older adults suggested that individuals with anemia did perform worse in SPPB test and muscle strength test than those without anemia [30]. A recent study of nonagenarians in Italy supported the association of low hemoglobin concentration with poor physical performance, even after adjustment for sociodemographic and health indicators [31].

Of note, we detected an interaction between high RDW and anemia on low SPPB scores, such that there was a linear relationship between a high RDW and low physical performance among individuals without anemia, but an inverted J-shaped relationship among individuals with anemia. These findings support the view that high RDW could be a valuable biomarker for impaired physical performance in older adults. A pathological increase in RDW probably precedes the onset of anemia and abnormality of other routine blood indicators [32], and thus, could be a sensitive biomarker of early anemia. The inverted J-shaped association between RDW and SPPB score among individuals with anemia suggested that a low RDW may also be biomarkers for poor physical function among older adults with anemia [33]. This was in agreement with reports from previous studies that showed the J-shaped association between RDW and mortality [34, 35]. Future follow-up studies will help further clarify their potential causal relationships and better understand possible mechanisms.

The mechanisms underlying the association of RDW with physical performance among older adults are not fully understood, but could be speculated. First, previous studies have shown that high RDW is closely related to inflammation and oxidative stress [36], which in turn could contribute to impaired physical performance [37]. Second, high RDW and anemia trigger hypoxia, affect regular oxygen supply to muscles, and further lead to physical dysfunction [38]. Third, RDW can be increased under the conditions of iron deficiency anemia, vitamin B_12_, folate deficiency anemia. Previous studies have suggested that iron deficiency could contribute to impairment in physical performance by obstructing the oxygen transport and mitochondrial metabolism [39]. Moreover, deficiencies of folate and vitamin B_12_ increase total homocysteine in circulation, which further leads to impairment of skeletal muscle strength [40] and gait speed [41].

The current study was the first large-scale population-based study that showed evidence for the association of an elevated RDW and anemia with poor performance in lower-extremity physical function among elderly people living in rural communities. However, our study also has limitations. First, a temporal relationship of abnormal RDW and anemia with poor physical performance cannot be determined owing to the cross-sectional nature of the study design. Second, the lack of relevant markers for etiological diagnosis and classification of anemia (e.g., serum ferritin, folate, vitamin B_12_, and erythropoietin) prevents us from more thoroughly exploring the relationships between RDW, subtypes of anemia (e.g., vitamin B_12_ or folate deficiency anemia, iron deficiency anemia, sickle cell anemia, and aplastic anemia), and physical function in older adults. This is highly relevant for understanding the potential mechanisms underlying the relationships of RDW, anemia, and physical function, which deserves further investigation. Finally, the study population was derived from only one rural area of western Shandong Province, characterized by relatively low income and no or very limited educational achievements. This should be kept in mind when generalizing our study findings to other populations.

## Conclusions

In conclusion, this cross-sectional study provided evidence supporting the association of an elevated RDW with poor performance in lower-extremity physical function among older people in rural China. We further revealed that a high RDW is linearly associated with poor lower-extremity physical function among people without anemia but an inverted J-shaped relationship in those with anemia. Future prospective follow-up studies are warranted to help clarify the temporal relationship of anemia and RDW to physical function and the relevance of RDW as a biomarker for predicting physical impairment in older adults. In addition, the potential mechanisms linking anemia and RDW with physical impairment or functional dependency as well as the preventive and therapeutic implications for delaying physical impairment and disability deserve further investigation.

## Data Availability

The datasets used and analysed during the current study are available from the corresponding author upon reasonable request and approval by the Steering Committee of MIND-China.
